# Earlier point-of-care ultrasound, shorter length of stay in patients with acute flank pain

**DOI:** 10.1186/s13049-022-01017-1

**Published:** 2022-04-21

**Authors:** Pei-Hsiu Wang, Jia-Yu Chen, Dean-An Ling, An-Fu Lee, Ying-Chih Ko, Wan-Ching Lien, Chien-Hua Huang

**Affiliations:** 1 Department of Emergency Medicine, National Taiwan University Hospital, No. 7, Chung-Shan S. Road, Taipei, 100 Taiwan; 2 Department of Emergency Medicine, College of Medicine, National Taiwan University, Taipei, Taiwan

**Keywords:** Point-of-care ultrasound, Length of stay, Emergency department, Flank pain

## Abstract

**Background:**

The effects of early integration of point-of-care ultrasound (PoCUS) into patient care are uncertain. This study aims to investigate the effects of early PoCUS on patients with acute flank pain.

**Methods:**

Adult non-traumatic patients with acute flank pain receiving PoCUS were enrolled. Expert physicians reviewed the medical records and made the “final diagnosis” for the cause of acute flank pain. The primary outcome was the relationship between the door to ultrasound (US) time and length of stay (LOS). The secondary outcomes included the sensitivity, specificity, positive predictive value (PPV), and negative predictive value (NPV) of the sonographic diagnosis, compared with the final diagnosis.

**Results:**

Eight hundred and eighty-eight patients were included in the analysis. Patients receiving early PoCUS (≤120 min) had a shorter LOS (128 *vs*. 217 min, *p* < 0.0001). Patients in the late POCUS group (> 120 min) had a trend to receive more CT scans. The disease distribution, sensitivity, specificity, PPV, and NPV were similar in patients receiving early or late PoCUS for target diagnoses. After adjusting for the confounders, early PoCUS (OR, 2.77, 95% CIs, 1.93–3.98) had a positive impact on shorter LOS. In addition, the effect of early PoCUS became more prominent (OR, 4.91, 95% CIs, 3.39–7.13) on LOS in less than 3 h.

**Conclusions:**

Early integration of PoCUS is significantly related to shorter LOS in patients with acute flank pain without increasing morbidity and mortality. Our results suggested “PoCUS early” in these patients to possibly alleviate emergency department crowding.

*Trial registration* NCT04149041 at the ClinicalTrial.gov.

## Background

Acute flank pain is a common symptom in the emergency department (ED). Acute ureteral obstruction from an impacted stone is the most frequent cause. However, a variety of diseases may manifest as acute flank pain, mimicking renal colic. Among them, pyelonephritis and myofascial pain are frequently encountered.

Besides a detailed medical history and physical examination, kidney-ureter-bladder (KUB) radiography and urinalysis are frequently used for the evaluation of acute flank pain although they exhibit a limited diagnostic value for urolithiasis [1, 2]. Currently, non-contrast computed tomography (CT) has become the gold standard for diagnosing acute flank pain, not only for urolithiasis but also for alternative diagnoses without the presence of the stone [3]. However, ionizing radiation and the costly expense of CT should be considered in emergency settings. A multicenter randomized trial demonstrated that initial point-of-care ultrasound (PoCUS) was associated with lower cumulative radiation exposure than initial CT. Notably, no significant differences in high-risk diagnoses, serious adverse events, and ED revisits between ultrasound (US) and CT [4]. However, the median length of stay (LOS) of patients remained more than 5 h in the PoCUS group, longer than the time targets (4 h) in emergency care [5].

With the advance of PoCUS, it can be used as an extension of the doctor’s hand to look inside and find out the possible etiology. The effects of early integration of PoCUS into actual patient care are uncertain. This study aims to investigate the effects of early PoCUS in the evaluation of ED patients with acute flank pain.

## Methods

### Study design and setting

The retrospective study was conducted at the ED of the National Taiwan University Hospital (NTUH), a tertiary medical center in Taiwan, between July 2015 and July 2017. The study protocol was approved by the Institutional Review Board of the Ethics Committee of the NTUH (201907173RIND) with a waiver of informed consent and registered at ClinicalTrials.gov (NCT04149041).

PoCUS was included in emergency residency US training since 2012, and all of the residents passed the hand-on assessment. The instructors were certified by the Taiwan Society of Ultrasound in Medicine and had more than 10 years of experience in sonographic examinations. All ultrasonographic examinations were written in a standard report form including indication, sonographic findings, sonographic diagnosis, and management.

Two US machines (SSA-550A, SSA-660, Canon, Japan) equipped with 2–5 MHz curvilinear transducers were set up and placed on standby for use.

### Patient enrollment

Adult non-traumatic patients (more than 20-year-old) presenting with acute flank pain referred for PoCUS were eligible. Patients aged less than 20 years, with pregnancy or trauma were excluded.

### Data collection

Clinical data were retrospectively obtained from the electronic medical records, including age, sex, body mass index (BMI), comorbidities, vital signs on arrival, time of visits, door to physician time, door to US time, door to KUB time, door to CT time, door to urinalysis time, ED LOS, and patient disposition, as well as the sonographic reports. The time of visits was categorized into weekday visits or weekend/holiday visits, as well as dayshift visits (8 a.m. to 8 p.m.) or nightshift visits (8 p.m. to 8 a.m.).

After patient discharge, expert ED physicians not involving PoCUS training reviewed the medical records and made the “final diagnosis” for the cause of acute flank pain (urolithiasis, pyelonephritis, myofascial pain, or others). The diagnostic criteria for urolithiasis included the presence of stones in the urinary tract in KUB, PoCUS, CT, or other imaging studies besides history and physical examination.

### Outcome measurement

The primary outcome was the relationship between the door to US time and LOS. The secondary outcomes included the sensitivity, specificity, positive predictive value (PPV), and negative predictive value (NPV) of the sonographic diagnosis, compared with the final diagnosis.

### Statistical analysis

All data were analyzed by SAS software (SAS 9.4, Cary, North Carolina, USA). Categorical data were expressed in counts and proportions and compared using a Chi-square test, while continuous data were expressed in medians and interquartile ranges (IQRs), and examined using Wilcoxon’s rank-sum test.

The linear regression model was applied to investigate the relationship between the door to US time and LOS. Covariates in the model included age, sex, BMI, comorbidities, time of visits, and door to physician time. Further, the patients were divided into two groups by the door to US time ≤120 min (early) or > 120 min (late). Between-group differences for the parameters were investigated.

The logistic regression model was applied to investigate the early use of PoCUS (≤120 min) associated with shortened LOS (≤240 min) [5]. Covariates in the model were age, sex, BMI, comorbidities, time to visits, door to physician time, and door to US time categorized by 120 min. Odds ratios (ORs) were computed with 95% confidence intervals (CIs) for significant parameters. Also, the model was re-built using LOS of 180 min as the cut-point. A p-value of less than 0.05 was considered statistically significant.

## Results

**Fig. 1:**
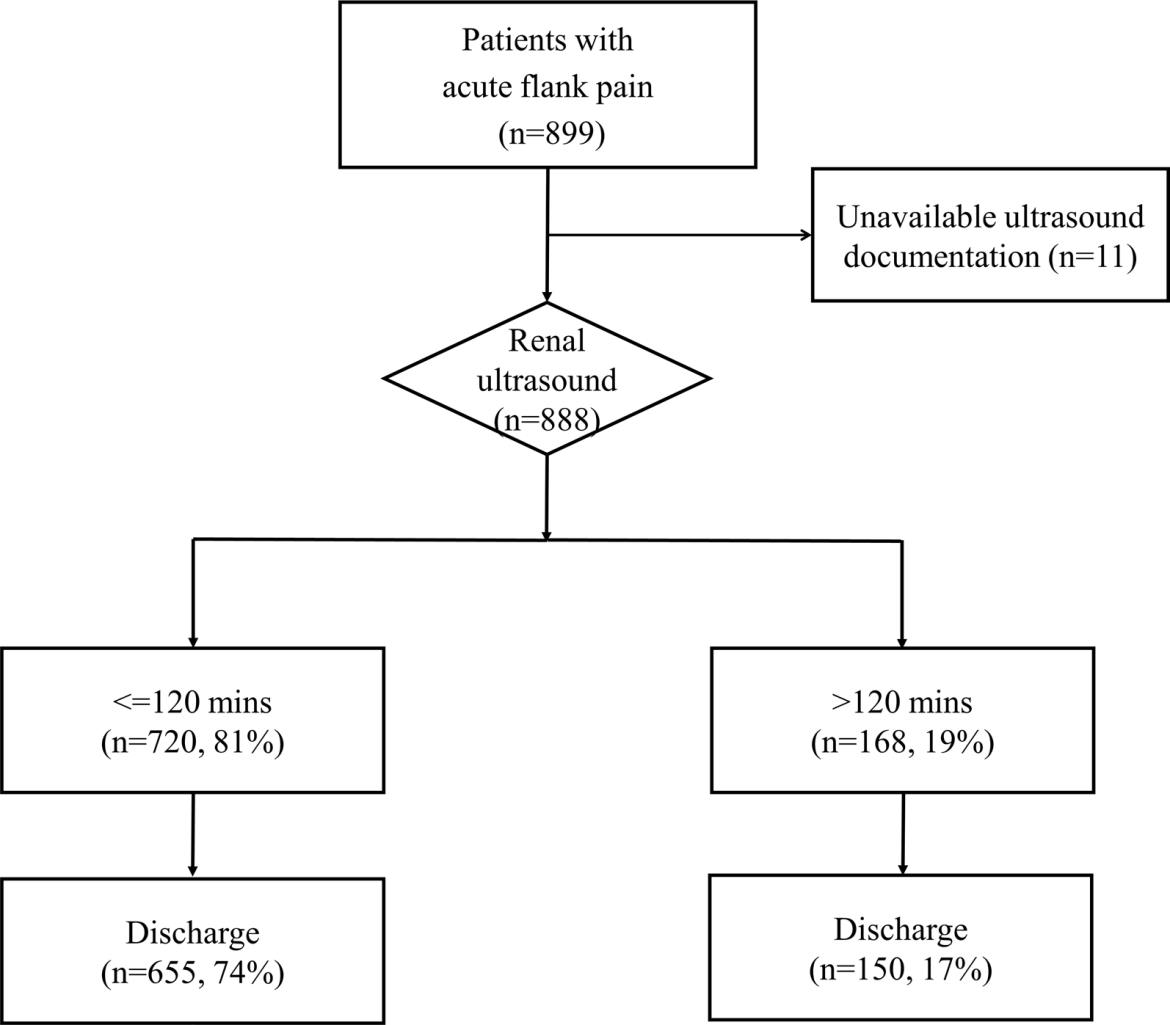
The study flowchart

During the study period, 899 patients with acute flank pain received PoCUS. After excluding 11 patients without available documentation, 888 patients were included in the analysis (Table 1, Fig. 1). The median age was 51 years (IQR, 39–62 years), and 495 (56%) were men. The median door to physician time, door to US time, door to urinalysis time and ED LOS were 19 (IQR, 16–23), 35 (IQR, 20–82), 59 (IQR, 42–90), and 146 (IQR, 100–255) minutes, respectively. Eight hundred and five patients (91%) were discharged from the ED. No mortality was observed.

**Table 1:** The characteristics of the included patients

	Total (n = 888)	PoCUS^†^≤120 min (n = 720)	PoCUS > 120 min (n = 168)	*p*-value^‡^
Age, years*	51(39, 62)	51 (40, 62)	52 (37, 61)	0.857
Male, n (%)	495 (55.7%)	396 (55.0%)	99 (58.9%)	0.356
Body mass index*	24.2 (21.7, 27.4)	24.1 (21.7, 27.3)	24.7 (21.7, 27.6)	0.211
Comorbidity				
Hypertension	190 (21.4%)	150 (20.8%)	40 (23.8%)	0.397
Diabetes mellitus	103 (11.6%)	84 (11.7%)	19 (11.3%)	0.896
Hyperlipidemia	72 (8.1%)	59 (8.2%)	13 (7.7%)	0.845
Chronic kidney disease	34 (3.8%)	28 (3.9%)	6 (3.6%)	0.847
Coronary artery disease	46 (5.2%)	33 (4.6%)	13 (7.7%)	0.097
Malignancy	59 (6.6%)	49 (6.8%)	10 (6.0%)	0.689
Vital signs				
Systolic blood pressure, mm-Hg*	141 (127, 159)	141 (126, 160)	142 (127, 155)	0.987
Diastolic blood pressure, mm-Hg*	80 (71, 90)	81 (71, 90)	80 (70, 90)	0.506
Heart rate, per min*	82 (72, 94)	82 (71, 94)	82 (73, 94)	0.639
SpO2, %*	98 (96, 99)	98 (96, 99)	98 (96, 99)	0.174
Weekend/holiday visits, n (%)	291 (32.8%)	230 (31.9%)	61 (36.3%)	0.278
Dayshift visits, n (%)	429 (48.3%)	349 (48.5%)	80 (47.6%)	0.842
Door to Physician time, min*	19 (16,23)	19 (16, 23)	21(16, 26)	0.068
Door to X-ray time, min*	39 (27, 59)	38 (27, 59)	43 (30, 61)	0.117
Door to US^†^ time, min*	35 (20, 82)	30 (18, 52)	168 (144, 237)	< 0.0001
Door to urinalysis time, min*	59 (42, 90)	60 (42, 89)	57 (43, 94)	0.267
Patients receiving CT^†^, n (%)	129 (14.5%)	97 (13.5%)	32 (19.0%)	0.065
Length of stay, min*	146 (100, 255)	128 (95,221)	217 (161,625)	< 0.0001
ED^†^ discharge, n (%)	805 (90.7%)	655 (91.0%)	150 (89.3%)	0.499

*Presented as median and interquartile ranges (IQRs)

^†^*US* ultrasonography, *CT* computed tomography, *ED* emergency department, *PoCUS* point-of-care ultrasound

^‡^Comparisons between patients with door to ultrasound time less than 120 min and more than 120 min

We investigated the relationship between the door to US time and LOS using a linear regression model. The results showed that the shorter the door to US time, the shorter the LOS (coefficient, 1.30 ± 0.14, *p* < 0.0001) after adjusting for age, sex, BMI, comorbidities, time of visits, and door to physician time.

Moreover, we categorized the patients into 2 groups based on the door to US time in less than 120 min (early) or more than 120 min (late) (Table 1). Among the patients, 720 patients received early PoCUS and the median door to US time was 30 (IQR, 18–52) minutes. The remaining 168 patients received late PoCUS, and the median door to US time was 168 (IQR, 144–237) minutes. Patients receiving early PoCUS had a shorter LOS (128 *vs*. 217 min, *p* < 0.0001). There were no significant differences in age, sex, body max index, comorbidities, vital signs on arrival, time of visits, door to physician time, door to x-ray time, door to urinalysis time, CT rate, and ED discharge rate between the two groups (Table 1). Patients in the late POCUS group had a trend to receive more CT scans.

Moreover, the final diagnoses for the cause of acute flank pain included urolithiasis (57%), acute pyelonephritis (15%), myofascial pain (14%), and miscellaneous diagnoses. There was no significant association between the door to US time and final diagnoses (Table 2). The patterns of disease distribution were similar among the groups receiving early or late PoCUS.

**Table 2:** The distribution of the target conditions

Diagnosis	POCUS^†^ ≤ 120 min (n = 720)	POCUS > 120 min (n = 168)	*p*-Value*
Urolithiasis, n (%)	403 (56.0)	100 (59.5)	0.403
Pyelonephritis, n (%)	100 (13.9)	30 (17.9)	0.190
Myofascial pain, n (%)	108 (15.0)	19 (11.3)	0.219
Others, n (%)	109 (15.1)	19 (11.3)	0.203
Cancer pain, n (%)	44 (6.1)	6 (3.6)	0.199
Functional GI^†^ disorders, n (%)	34 (4.7)	5 (3.0)	0.320
Renal hemorrhage, n (%)	2 (0.3)	0	0.494
Abdominal aortic aneurysm, n (%)	3 (0.4)	1 (0.6)	0.756
Gallstones, n (%)	2 (0.3)	1 (0.6)	0.523
Herpes zoster, n (%)	1 (0.1)	1 (0.6)	0.261
Polycystic kidney disease, n (%)	1 (0.1)	1 (0.6)	0.261
Miscellaneous, n (%)	21 (2.9)	4 (2.4)	0.705

*Comparisons between patients with door to renal ultrasound less than 120 min and more than 120 min

^†^*POCUS* point-of-care ultrasound, *GI* gastrointestinal

Furthermore, the logistic regression model was applied to investigate the factors associated with LOS less than 4 h. After adjusting for the confounders, early PoCUS (OR, 2.77, 95% CIs, 1.93–3.98) had a positive impact on shorter LOS. By contrast, patients with malignancy (OR, 0.54, 95% CIs, 0.30–0.98) had longer LOS. In addition, the effect of early PoCUS became more prominent (OR, 4.91, 95% CIs, 3.39–7.13) on LOS in less than 3 h.

The sensitivity, specificity, PPV, and NPV were similar in patients receiving early or late PoCUS for target diagnoses (Table 3). The presence of unilateral hydronephrosis on PoCUS was associated with high sensitivity, specificity, PPV, and NPV for the diagnosis of urolithiasis, whether in early or late PoCUS groups. However, normal kidneys (i.e. without the presence of hydronephrosis or other specific findings) in the sonographic finding had varying sensitivity and specificity for the diagnosis of pyelonephritis and myofascial pain.

**Table 3:** The sensitivity, specificity, positive predictive value, and negative predictive value of the sonographic findings for target diagnoses

	Sonographic finding	PoCUS^†^ time (min)	Sensitivity^‡^, %	Specificity^‡^, %	PPV^†‡^, %	NPV^†‡^, %
Urolithiasis (n = 503)	Unilateral hydronephrosis	≤120	86.4 (83.0,89.7)	83.0 (78.8,87.1)	86.6 (83.2,89.9)	82.7 (78.6,86.9)
		> 120	86.0 (79.2,92.8)	76.5 (66.4,86.6)	84.3 (77.3,91.4)	78.8 (68.9,88.7)
Pyelonephritis (n = 130)	Normal kidney*	≤120	63.0 (53.5,72.5)	69.4 (65.7,73.0)	24.9 (19.6,30.2)	92.1 (89.6,94.5)
		> 120	56.7 (38.9,74.4)	74.6 (67.4,81.9)	32.7 (19.9,45.4)	88.8 (83.1,94.5)
Myofascial pain (n = 127)	Normal kidney*	≤120	100 (100,100)	77.5 (74.1,80.8)	43.9 (37.7,50.1)	100 (100,100)
		> 120	100 (100,100)	77.9 (71.2,84.5)	36.5 (23.5,49.6)	100 (100,100)

*Indicated without the presence of hydronephrosis or other specific findings

^†^*PoCUS* point-of-care ultrasound, *PPV* positive predictive value, *NPV* negative predictive value

^‡^() indicates 95% confidence intervals

## Discussion

Acute flank pain is a frequently encountered complaint at the ED. Compared with non-contrast CT, US has the advantage of bedside accessibility and a lack of ionic radiation. Our study investigated the effect of PoCUS on patient-centered outcomes. The results showed that the earlier use of PoCUS was associated with shorter ED LOS in patients with acute flank pain. An earlier PoCUS did not harm the diagnostic accuracy including sensitivity, specificity, PPV, and NPV.

Urolithiasis is the most frequent cause among patients with acute flank pain. Previous studies have suggested that US is the ideal initial imaging test in the ED setting for patients with suspected urolithiasis without changing patient outcomes and morbidity [4, 6]. Modern ED provided not only urgent care for life-threatening emergencies but also further diagnostic assessment and workups [7]. Thus, ED LOS was substantially increasing, which resulted in ED crowding and subsequent complications, and increased mortality [8, 9]. Moreover, a decrease in ED boarding and crowding would prevent in-hospital transmission and infection during the global COVID-19 pandemic. Our results well demonstrated that early integration of PoCUS in clinical practice was significantly associated with lessening ED LOS, possibly alleviating ED crowding. Also, the effect of early PoCUS remained significant after adjusting the door to physician time and other confounders, implying it was not associated with early or late access to the physician.

There have been still some concerns regarding the use of PoCUS for the diagnosis of urolithiasis. PoCUS has limited ability to visualize stones less than 3 mm [10], or in the mid-ureter due to the interference of overlying bowel gas. Also, a high BMI would result in unclear US images and restricted fields of view. On the other aspect, a small subset of patients with tiny ureteral stones does not develop hydronephrosis indeed. However, even in patients with severe hydronephrosis, permanent damage to the kidneys may only occur after 2 weeks; therefore, emergency interventions may not be required [11]. In this study, we adopted hydronephrosis as an indirect sign of urolithiasis, and the results presented with acceptable sensitivity and specificity. Also, the discharge rate was more than 90%, in agreement with a previous review [12]. Moreover, no adverse outcomes were observed.

In recent years, PoCUS has become a required milestone competency in residency practice [13–16]. PoCUS can be considered a “21st-century stethoscope” to evaluate a broad spectrum of illnesses and change the management of a wide variety of patients in emergent settings [17, 18]. A well-established training program with an easily accessible US machine is essential for effective incorporation into clinical care. However, previous studies mainly focused on the diagnostic accuracy of PoCUS compared with CT or US performed by radiologists [4, 10], rarely on how to integrate PoCUS during clinical practice. Our study provided evidence that PoCUS could be a part of the timely evaluation of acute flank pain.

Moreover, diseases of the abdominal aorta represent a category of life-threatening conditions, which present with flank pain [19]. In our study, 4 cases were diagnosed to have abdominal aortic aneurysms. Of them, 3 (75%) were diagnosed with early PoCUS, although there was no significant difference in diagnosing aortic aneurysms between early and late PoCUS. However, timely recognition and proper management would be beneficial to these patients to reduce morbidity and mortality [20].

There were limitations in this study. First, because our study was a retrospective design, some US images and records were missing. However, the missing data was only 1% and the results were representative of the large-scale study. Second, there were no absolute guidelines for how early to use US in patients with acute flank pain. It depended on physicians’ clinical discretions, which may result in selection bias. However, the distribution of etiologies was similar in patients receiving US in 120 min or later. Also, the diagnostic accuracy of PoCUS was similar between these two groups. Third, the factors influencing the LOS were multifactorial, occurring in the input, throughput, and output [21], although we had adjusted the selected covariates in the regression model. However, other factors which existed at the same time of patient visits such as already crowding, busy ED, or different doctors were hard to be explored due to a retrospective design. Fourth, we adopted a “normal” sonographic finding for the diagnosis of pyelonephritis or myofascial pain. Notably, a normal finding does not exclude the presence of other diseases, and the physician should correlate the sonographic finding into clinical context/supplemental tests. Last, our hospital was a teaching hospital where the comorbidity and disease patterns would be more severe and complicated. As the results shown in this study, a substantial percentage of patients presented with flank pain due to existing malignancies and exhibited a longer LOS. Also, someone would wonder if the longer LOS was a result of diagnostic uncertainty due to a less "typical" presentation. However, the disease patterns and the percentage of discharge were similar between the early and late PoCUS groups.

## Conclusions

Early integration of PoCUS for ED patient care is significantly related to shorter LOS in patients with acute flank pain without increasing morbidity and mortality. Our results suggested “PoCUS early” in these patients to possibly alleviate ED crowding. However, many factors are involved in ED patient flow that further investigations should be explored.

## Data Availability

All data analyzed during this study are included in this published article.

## Abbreviations

ED: Emergency department
KUB: Kidney-ureter-bladder
CT: Computed tomography
PoCUS: Point-of-care ultrasound
US: Ultrasound
LOS: Length of stay
NTUH: National Taiwan University Hospital
BMI: Body mass index
PPV: Positive predictive value
NPV: Negative predictive value
IQR: Interquartile range
OR: Odds ratio
CI: Confidence interval
